# Composition and Organization of Acute Ischemic Stroke Thrombus: A Wealth of Information for Future Thrombolytic Strategies

**DOI:** 10.3389/fneur.2022.870331

**Published:** 2022-07-06

**Authors:** Jean-Philippe Desilles, Lucas Di Meglio, Francois Delvoye, Benjamin Maïer, Michel Piotin, Benoît Ho-Tin-Noé, Mikael Mazighi

**Affiliations:** ^1^Interventional Neuroradiology Department and Biological Resources Center, Rothschild Foundation Hospital, Paris, France; ^2^Laboratory of Vascular Translational Science, U1148 INSERM, Paris, France; ^3^Université Paris Cité, Paris, France; ^4^FHU Neurovasc, Paris, France; ^5^University of Liège, Liege, Belgium; ^6^Department of Neurology, Hopital Lariboisère, APHP Nord, Paris, France

**Keywords:** ischemic stroke, thrombus composition, fibrin, platelet aggregates, neutrophil extracellular traps (NETs), von Willebrand factor (vWF)

## Abstract

During the last decade, significant progress has been made in understanding thrombus composition and organization in the setting of acute ischemic stroke (AIS). In particular, thrombus organization is now described as highly heterogeneous but with 2 preserved characteristics: the presence of (1) two distinct main types of areas in the core—red blood cell (RBC)-rich and platelet-rich areas in variable proportions in each thrombus—and (2) an external shell surrounding the core composed exclusively of platelet-rich areas. In contrast to RBC-rich areas, platelet-rich areas are highly complex and are mainly responsible for the thrombolysis resistance of these thrombi for the following reasons: the presence of platelet-derived fibrinolysis inhibitors in large amounts, modifications of the fibrin network structure resistant to the tissue plasminogen activator (tPA)-induced fibrinolysis, and the presence of non-fibrin extracellular components, such as von Willebrand factor (vWF) multimers and neutrophil extracellular traps. From these studies, new therapeutic avenues are in development to increase the fibrinolytic efficacy of intravenous (IV) tPA-based therapy or to target non-fibrin thrombus components, such as platelet aggregates, vWF multimers, or the extracellular DNA network.

## Introduction

Until recently and the emergence of endovascular therapy (EVT), little was known about thrombus composition and their histological characteristics in acute ischemic stroke (AIS). However, such information might be of the utmost value to better understand the mechanisms involved in thrombolysis resistance. For several years now, EVT has become the standard-of-care for AIS patients with large vessel occlusion, along with a growing number of available thrombus samples and stimulating research to evaluate thrombus microscopic features. Two previous reviews nicely provided an overview of the current knowledge and expanding research relating to histological features of AIS thrombus (1, 2). The importance of studying thrombus composition and structure is stressed by recent findings that indicate that targeting fibrin and optimizing its lysis by tissue plasminogen activator (tPA) should not be the sole strategy for improved thrombolysis. In fact, at least three *in vitro* studies conducted by different groups have provided converging evidence that thrombi can persist and continue growing during the recruitment of platelets despite ongoing and effective tPA-mediated fibrinolysis (3–5). These findings demonstrate that resistance to thrombolysis does not necessarily equal resistance to fibrinolysis and suggest that degrading thrombus components other than fibrin might be required for optimal and durable thrombolysis. Notably, a clinical study found that arterial re-occlusion can occur even after successful recanalization induced by intravenous (IV) tPA (6). Consistent with those results, accumulating evidence indicates that drugs aimed at disrupting extracellular DNA (7–9), platelet aggregates (3, 10, 11), or von Willebrand factor (vWF) multimers (12, 13) could help in the design of new thrombolytic strategies with increased efficacy. Of note, two recently published studies focusing on AIS thrombus structure and composition have provided evidence that such non-fibrin components are particularly present in tPA-resistant areas of AIS thrombi (14, 15).

The translational studies based on the analysis of mechanically retrieved thrombi share a common limitation because based on removed thrombi, excluding both dissolved thrombi (either spontaneously or after IV tPA) and thrombi impossible to retrieve. For this reason, our review will present the recent data on what we have learned about thrombolysis-resistant thrombi from the analysis of EVT-recovered thrombi to discuss future therapeutic avenues.

## Thrombus Organization and Ultrastructural Features

**Figure 1:**
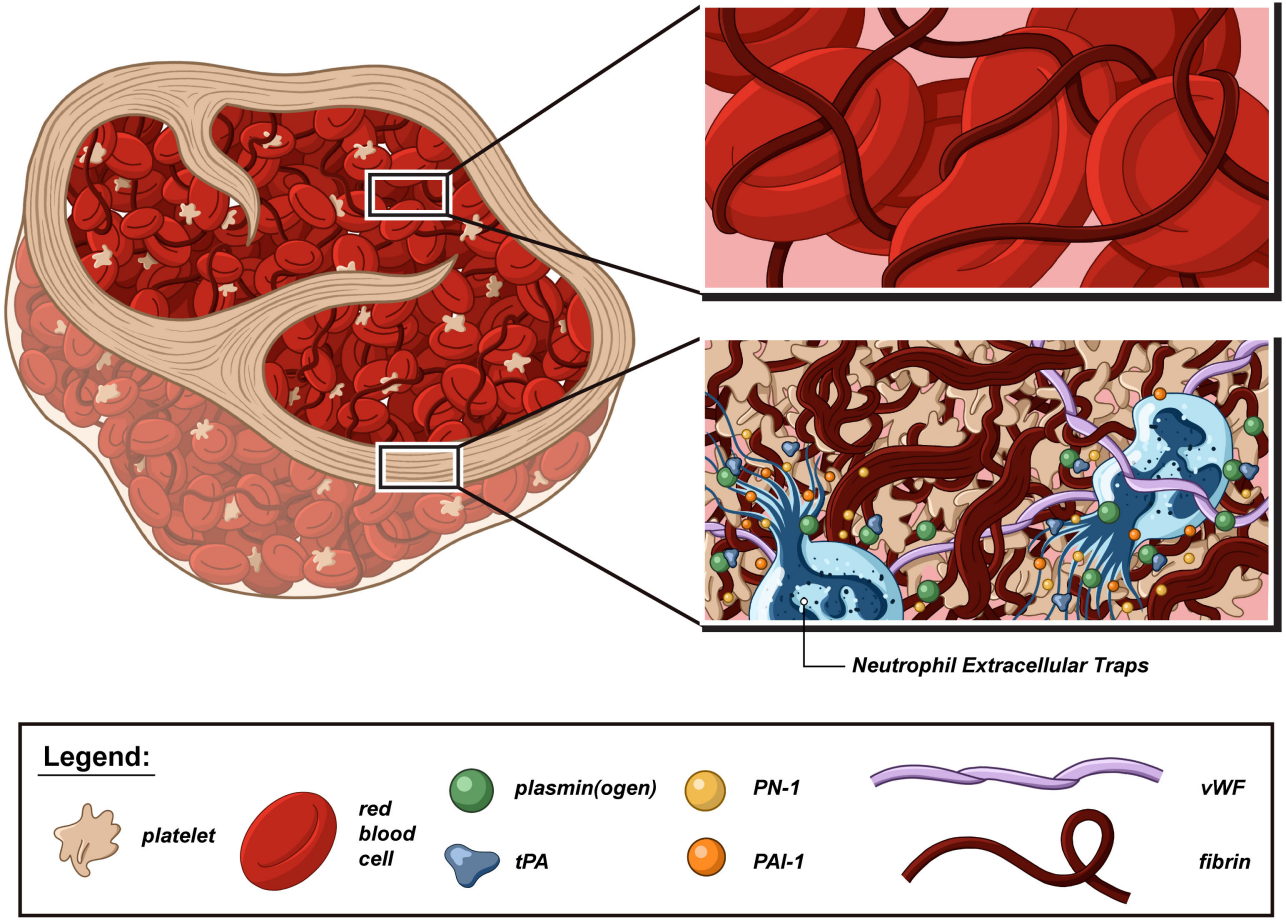
Schematic representation of a thrombolysis resistant acute ischemic stroke thrombus. Although acute ischemic stroke (AIS) thrombi are highly variable in size, shape, and cell proportion, they share the same basic fibrillar and cellular components, and organization domains. There is converging evidence that platelets, neutrophils, von Willebrand factor (vWF), and neutrophil extracellular traps (NETs) form thrombolysis-resistant microdomains that are notably characterized by a higher degree of fibrin compaction and increased content in fibrinolysis inhibitors, such as plasminogen activator inhibitor-1 (PAI-1) and protease nexin-1 (PN-1). In contrast, the fibrin network is looser in red blood cell-rich areas and can be further loosened *via* the combined action of tissue-type plasminogen activator (tPA) and plasminogen.

Di Meglio et al. (14) and Autar et al. (16) showed preserved organization of AIS thrombi with the presence of an external shell surrounding a core of heterogeneous aspect (Figure 1). This shell was particularly visible in thrombi whose inner core was rich in red blood cells (RBCs). Especially, thrombus analysis by scanning electron microscopy revealed the presence of a dense, sealed external shell encapsulating a loose RBC-rich core in most thrombi. In platelet-rich thrombi, the inner core can sometimes be “shell-like” (14). The shell components were so densely compacted and agglomerated that they form a continuous layer in which individual cells could hardly be detected. This observation was in stark contrast to the clearly identifiable RBCs, fibrin fibers, and aggregated platelets in the inner core. Immunofluorescent staining analyses also showed that this outer shell consisted of densely compacted thrombus components that included fibrin(ogen), vWF multimers, and aggregated platelets, with a clear difference between the surface and inner core organization of AIS thrombi. In the shell, the fibrin network appeared to be sealed in a uniform layer. In contrast, in the thrombus core, the network appeared looser, with gaps interposed by RBCs. Immunostaining of AIS thrombi for platelet-derived direct inhibitors of tPA, plasminogen activator inhibitor 1 (PAI-1), and protease-nexin 1 (PN-1) showed that both PAI-1 and PN-1 were abundant and preferentially accumulated in the platelet-rich outer shell (14). These results are in accordance with a previous *in vitro* study showing that during thrombus formation, fibrin and platelet aggregates were redistributed on the surface of the contracted thrombus with a thick fibrin meshwork, with few platelet aggregates in the core, which consisted mostly of compacted RBCs (17). To test the hypothesis that the external layer provides a protective coating against thrombolysis, the rate of *in vitro* thrombolysis was compared when thrombi were left intact or cut in half in order to breach their shell and expose their inner core. The rate of thrombolysis was significantly lower for intact thrombi than for those with a breach in their external shell (14). Interestingly, comparisons of thrombi recovered from patients having received IV tPA or not prior to EVT indicated that IV tPA-induced fibrinolysis may facilitate EVT by altering thrombus structure. In particular, IV tPA was reported to cause a significant loosening and thinning of the fibrin network, with a grid- and sponge-like aspect, which resulted in a reduced compaction of RBCs and, presumably, in a more porous structure (18, 19). Another interesting study reported that sonothrombolysis might affect the tPA potency by changing thrombus organization and properties (20).

## Thrombus Composition and Internal Organization

**Figure 2:**
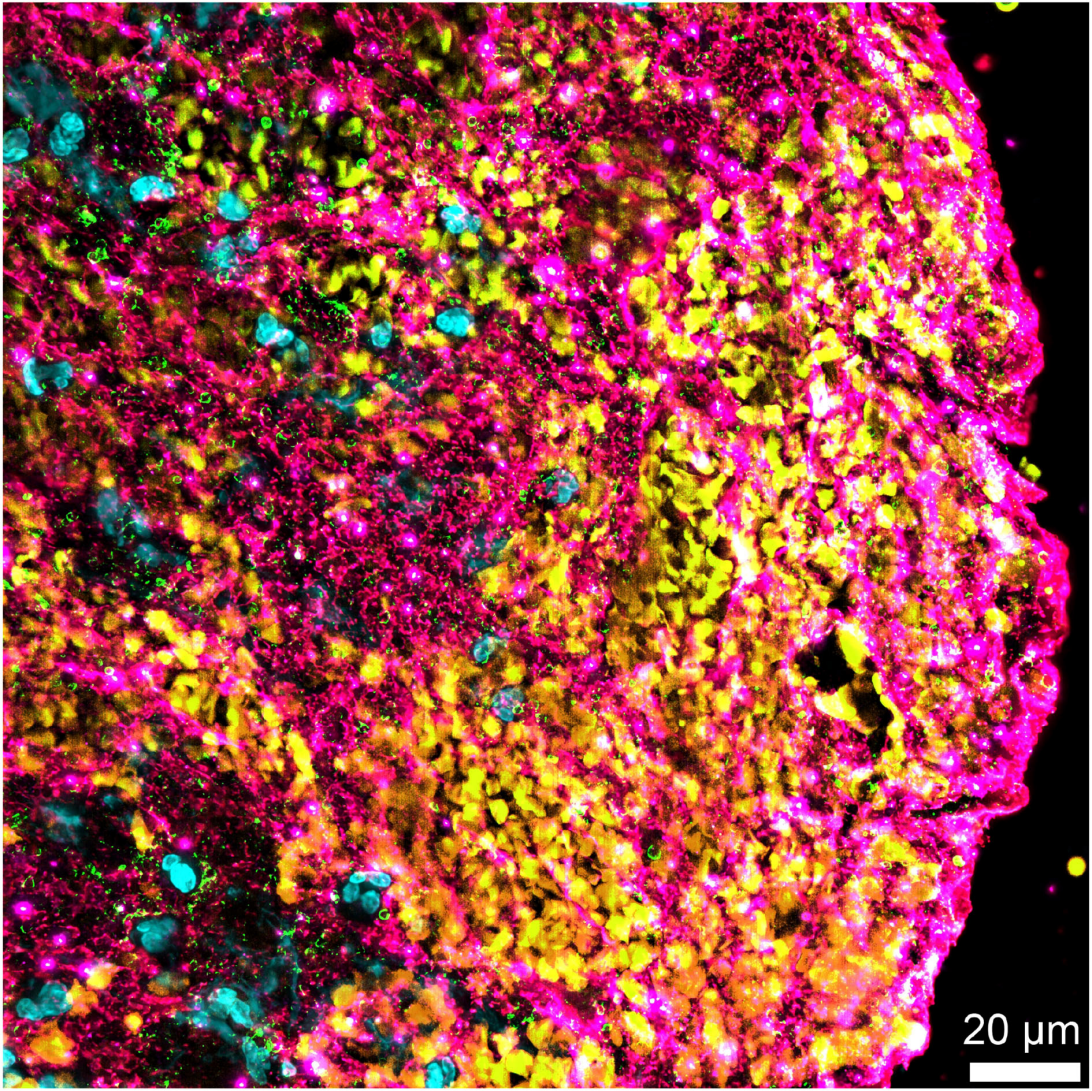
Non-fibrin components in an acute ischemic stroke thrombus. Immunofluorescent staining of an acute ischemic stroke (AIS) thrombus for DNA (Hoechst 33342, blue), platelet glycoprotein Ib (GPIb; green), red blood cells (yellow), and von Willebrand factor (vWF) (pink) showing the abundance of non-fibrin components, several of which are potential pharmacological targets for improved thrombolysis.

A recent immunostaining study analyzed the organization and repartition of different components of a large series of AIS thrombi (15). Thrombi were found to be composed of two main types of areas: RBC-rich areas and platelet-rich areas. RBC-rich areas had limited complexity because they consisted of RBCs entangled in a meshwork of thin fibrin strands. In contrast, platelet-rich areas were highly complex and characterized by dense fibrin structures aligned with vWF multimers and with abundant amounts of neutrophils and neutrophil extracellular traps (NETs) accumulated around them. These latter platelet-rich areas were supposed to be involved in much of the thrombolysis resistance, and their components were specifically studied (Figure 2).

### The Fibrin Network and Its Modifications

The susceptibility of the fibrin network to lysis is dictated by its structure (21). Several post-translational modification processes were found responsible for fibrin network maturation and fibrinolysis resistance. Post-translational modifications are (bio)chemical reactions in which amino-acid residues of proteins or peptides are covalently modified (22). This process significantly increases the structural and functional diversity of proteins, thus introducing a complexity to the proteome (23). Concomitant with converting fibrinogen to fibrin, thrombin activates transglutaminase factor XIII. Activated factor XIII (factor XIIIa) contributes to the conversion of the initially loose fibrin network into the firm insoluble polymer capable of providing biophysical and biochemical support to the thrombus (24). Factor XIIIa also inhibits fibrinolysis by forming fibrin–α2 anti-plasmin cross-links that prevent this plasmin inhibitor from being fully expelled from the thrombus during compaction/retraction (25). Like α2 anti-plasmin, PAI-2, which is derived from macrophages, can also be cross-linked to fibrin by factor XIIIa (26). An *in vitro* study found that under experimental conditions that increased the rate of non-enzymatic protein glycosylation, fibrin networks were associated with correspondingly greater degrees of resistance to degradation by plasmin (27). As noted above, the fibrin network found at the surface of AIS thrombi showed an altered morphology. Fibrin in the shell of AIS thrombi indeed displays a sealed aspect that strikingly resembles that of fibrinolysis-resistant, dense, matted fibrin deposits produced by various modifications of fibrin, such as oxidation (28), carbamylation (29), and exposure to platelet factor 4 (30) or air (31). Together, these findings indicate that in addition to factor XIIIa-mediated cross-linking of the fibrin network, other chemical modifications likely help to increase the resistance of AIS thrombi to fibrinolysis.

In addition to chemical modifications, mechanical compaction and/or deformation also affect the susceptibility of the fibrin network to tPA-mediated fibrinolysis. Thrombus contraction indeed impairs external fibrinolysis by limiting thrombus permeability and thus the accessibility of fibrin fibers to fibrinolytic agents (25). Of note, platelets, which are essential for thrombus retraction, were recently found required for AIS thrombus shell formation (14). Thus, the compaction of fibrin fibers with other thrombus components in the shell of AIS thrombi might result from a platelet-mediated mechanical effect.

### Platelet Aggregates

Platelets are crucial to the fibrinolysis response (32). Platelet aggregation plays a central role in thrombus formation, as an anchor for the initiation and growth of the thrombus scaffold. Interaction of platelets with the exposed components of the injured or activated vessel wall generates the initial plug. When platelets bind matrix proteins or activated endothelial cells, they undergo rapid activation to release coagulation factors into the surrounding milieu. Upon activation, platelets release more than 300 proteins, most arising from their secretory α-granules. Several fibrinolytic inhibitors exist within platelet α-granules and as a result, platelet-rich thrombi are reportedly more resistant to tPA-mediated lysis than are RBC-rich thrombi (33). PAI-1 is abundant in α-granules and is released upon platelet activation, accounting for most of the circulating PAI-1 (34). In addition, platelets change both the shape and composition of their membrane, which allows for direct interaction with coagulation proteins, circulating fibrinogen and neutrophils. Finally, platelets undergo a reorganization of their cytoskeleton, mainly because of activation of the fibrin–αIIbβ3–myosin axis allowing them to exert a contractile force on extracellular fibrin fibers responsible for thrombus retraction (35). As compared with non-retracted thrombi, retracted thrombi are more resistant to fibrinolytic degradation (36) and lyse more slowly (37). In an experimental model of embolic stroke in rats, platelet-rich thrombi resulted in high rates of middle cerebral artery occlusion, low rates of spontaneous thrombolysis, and large infarction. These platelet-rich thrombi were highly resistant to tPA (38). A clinical study found that patients with platelet-rich thrombi had poorer revascularization outcomes than those with RBC-rich thrombi. Platelet and vWF levels in AIS thrombi were correlated with each other and both were inversely related to the amount of RBCs (39).

### Neutrophils, NETs, and Extracellular DNA

Neutrophil extracellular traps are fibrous networks of extracellular DNA released by neutrophils in response to stimuli; they are in the form of decondensed chromatin associated with citrullinated histones and neutrophil granule proteins (40). In recent years, the important contribution of neutrophils, particularly *via* their NETs, to thrombus pathogenesis has been increasingly recognized (41, 42). NETs provide a physical scaffold for thrombus growth by binding platelets and RBCs (43). Recent immunostaining analyses found that every AIS thrombus contained NETs. A study found a significantly higher amount of NETs in AIS thrombi from a cardiac than non-cardiac origin (8). NETs were predominantly located in platelet-rich areas that include the outer layer of AIS thrombi (7, 15). In *ex vivo* thrombolysis assays, tPA and deoxyribonuclease 1 (DNAse 1) incubation accelerated thrombolysis as compared with tPA or DNAse 1 alone (7, 8). More recently, we confirmed in a new *ex vivo* study that the ability of DNAse 1 to improve tPA-mediated thrombolysis was linked to a potentiation of fibrinolysis, as indicated by increased release of plasmin-generated D-dimers in the presence of DNAse 1 (44). These data confirm that NETs contribute to thrombus composition regardless of their origin and participate in fibrinolysis resistance.

Regarding resistant thrombi to EVT, those are rare, both in our experience and in large recent series of EVT-treated patients, with proximal recanalization rates now nearing 90% (45). Data on the characteristics of those remaining 10% proximal thrombi that are resistant to both thrombolysis and EVT are scarce. Nonetheless, information on this thrombus category may be extrapolated from analyses of thrombi that were particularly difficult to retrieve mechanically. NET thrombus content was found to be positively correlated with endovascular procedure length and device number of passes (7). In a recent case report by Staessens et al., the authors described the histological features of a thrombus that had required 11 attempts to be retrieved mechanically. This nearly EVT-resistant thrombus was characterized by a core that was particularly rich in extracellular DNA, vWF, and microcalcifications (46). With the exception of the presence of microcalcifications, comparable observations were made by Abbasi et al. who reported increased levels of NETs and vWF in thrombi with delayed time to recanalization, as compared to thrombi with early recanalization time (47). Considering that longer recanalization delay is associated with a reduced recanalization success rate, the results from these recent studies converge to support the hypothesis that NETs, vWF, and extracellular DNA with microcalcifications favor EVT resistance.

### vWF Multimers

von Willebrand factor is a large multimeric plasma glycoprotein and plays a crucial role in arterial thrombus formation. Particularly, vWF recruits platelets at sites of vascular injury by acting as a molecular bridge between circulating platelets and exposed components of the subendothelium (48). Together with fibrin(ogen), vWF links platelets together, which further stabilizes platelet aggregates. High vWF level and low level of a disintegrin-like, metalloprotease thrombospondin type 1 motif, and member 13 (ADAMTS13) in plasma were identified as important risk factors for AIS (49, 50). After immunostaining analysis of AIS thrombi, one study found 20% vWF content, which was inversely correlated with thrombus RBC content (12). In addition, reduced plasma ADAMTS13 activity was associated with poor response to recanalization therapies (51).

## New Therapeutic Avenues

A first strategy could be to increase the fibrinolytic efficacy of IV tPA-based therapy. A second strategy consists of targeting non-fibrin AIS thrombus components, such as platelet aggregates, vWF multimers, or the extracellular DNA network (Table 1).

**Table 1:** Summary of the potential treatment approaches, their mechanisms of action, and ongoing clinical development.

**Thrombus component target**	**Molecule**	**Mechanism of action**	**Ongoing clinical trials**
**Fibrin network**	Tenecteplase	Genetically modified form of human tPA	Several randomized phase 3 trials are ongoing to confirm the efficacy of tenecteplase compared to alteplase (NCT03854500; NCT04915729)
	TAFI inhibitor	Inhibitor of fibrinolysis inhibitor	Two ongoing phase 1–2 clinical trials are assessing the safety of a TAFI inhibitor infusion (NCT03198715; NCT02586233)
	PAI-1 inhibitor	Inhibitor of fibrinolysis inhibitor	None
**vWF multimers**	*N*-acetylcysteine (NAC)	Reducing intrachain disulfide bonds in large polymeric proteins	The NAC for thrombolysis in acute stroke (NAC-S) trial (ClinicalTrials.gov: NCT04920448) is an ongoing phase 2 randomized study assessing the safety of NAC combined with IV tPA in AIS setting
	rhADAMTS-13	Specific VWF-cleaving metalloprotease	None
**Platelet aggregates**	Cangrelor	Platelet P2Y12 receptor inhibitor	A phase 3 randomized trial is currently assessing the benefit of cangrelor infusion in imaging-selected AIS patients eligible for EVT (ClinicalTrials.gov: NCT04667078)
	Glenzocimab	Antibody to platelet glycoprotein VI	Phase 2 randomized trial is currently assessing the safety of glenzocimab infusion on top of IV tPA in AIS patients (ClinicalTrials.gov: NCT03803007)
**Extracellular DNA**	Dornase Alfa	Recombinant human form of DNAse 1	A pilot phase 2 trial is assessing the safety and efficacy of IV DNase 1 in AIS patients receiving IV tPA and eligible for EVT (NETs-TARGET study, ClinicalTrials.gov: NCT04785066)

## Increasing the Fibrinolysis Efficacy of IV tPA

### Modified tPA

Tenecteplase is a genetically modified form of human tPA. These mutations confer 15-fold higher fibrin specificity than the recombinant human form of tPA (alteplase) and reduced binding to PAI-1, which leads to greater resistance to its inactivation (52). In patients with AIS, tenecteplase consumed less plasminogen and fibrinogen than did alteplase (53). Tenecteplase is also associated with an ~10-fold prolonged plasma half-life when compared with alteplase (52). This new pharmacokinetic property allows for a unique IV bolus injection when compared with the continuous 1-h infusion required with alteplase therapy. Of note, AIS trials suggest that tenecteplase may be associated with lower hemorrhagic transformation (HT) risk than alteplase but with a similar or superior recanalization rate (54). A randomized trial revealed that IV tenecteplase before EVT was associated with a significantly higher incidence of reperfusion and better functional outcome than with IV alteplase (55). Several international randomized clinical trials are still ongoing to confirm this superiority of tenecteplase over alteplase. The last US and EU guidelines stipulate that tenecteplase might be considered an alternative to alteplase before EVT (56, 57).

### Functionalized Administration of tPA

To date, only a few preclinical studies have explored this therapy. Nanomedical approaches for local or targeted delivery of thrombolytic drugs have aroused growing interest (58). However, more *in vivo* studies are needed to consider this strategy in the future.

### Fibrinolysis Inhibitors

Targeting anti-fibrinolytic proteins to boost endogenous fibrinolysis, raise the success of plasminogen activators, and lower the required therapeutic dose constitutes a promising approach for improving AIS treatment (59).

In the past three decades, many research groups and pharmaceutical companies have invested in the development of fibrinolysis inhibitors, such as mainly thrombin-activated fibrinolysis inhibitor (TAFI) and/or PAI-1 inhibitors. However, the question is still whether fibrinolysis inhibitors could be considered a new treatment strategy for improving fibrinolysis.

Several experimental studies showed a clear benefit of TAFI and/or PAI-1 inhibition but mainly in transient occlusion models of AIS (60–62). This impact was induced by both reduced microvascular thromboinflammation and better collateral perfusion. In a thromboembolic model of AIS, a TAFI inhibitor in association with reduced tPA dosage was associated with reduced ischemic lesion growth as compared to full tPA dosage alone. In this study, the TAFI inhibitor alone had no impact (63).

Two ongoing phase 1–2 clinical trials are assessing the safety of a TAFI inhibitor infusion. The first is recruiting non-selected patients with AIS and the second one focuses on AIS treated by EVT. Both have a primary end point of safety (ClinicalTrials.gov: NCT03198715). To date, to our knowledge, no PAI-1 inhibitor has been tested clinically.

Besides PAI-1, PN-1 represents another platelet-derived serpin with the ability to inhibit tPA-mediated fibrinolysis (64). Inhibitory single-domain antibodies to PN-1 were recently developed and might offer an additional therapeutic option to potentiate fibrinolysis (65). Nonetheless, as compared to PAI-1 inhibition, PN-1 inhibition might be associated with an increased risk of adverse effects, because PN-1 is a dual serpin that limits not only fibrinolysis but also thrombosis (66).

### Factor XIIIa Inhibition

As specified previously, factor XIIIa plays a crucial role in thrombus stability and could represent an interesting target to improve the tPA-induced thrombolysis. In this perspective, a recent preclinical study using a murine model where fibrin cross-linking by factor XIIIa was reduced demonstrated that this strategy might be associated with an increased risk of thrombus fragmentation and embolization without affecting thrombus size or its susceptibility to lysis (67).

## Targeting Non-Fibrin Contents of Thrombi

Obviously, the fibrin network is not the only target in an AIS thrombus. As discussed previously, the thrombus scaffold is also formed with platelet aggregates, vWF, and NET networks, so these latter components are promising new targets for future therapies on top of tPA or perhaps instead of it.

### Platelet Aggregates

Emerging experimental and clinical data demonstrate that platelets have a dual role in the setting of AIS. Undeniably, platelets play a key role in thrombus formation and resistance to thrombolysis. Nevertheless, platelets are also essential to maintain vascular integrity and prevent HT after AIS.

In the early 2000s, two clinical trials failed to demonstrate the benefit of αIIbβ3 inhibitors alone (abciximab and tirofiban) in AIS. In contrast, these trials showed that αIIbβ3 inhibitor infusion increased the HT rate, with significantly worse neurological outcomes (68, 69).

A still growing number of studies are highlighting potentially important differences in the pathways mainly involved in platelet aggregation in physiological hemostasis vs. pathological thrombosis. These studies raise the prospect of developing new antiplatelet drugs that specifically target mechanisms in thrombosis with a low risk of bleeding because they are dispensable for hemostasis. Platelet glycoprotein VI is a typical example of a platelet pathway that is dispensable for physiological hemostasis but critical for thrombus formation and growth. A therapeutic antibody to platelet glycoprotein VI, glenzocimab, was recently tested in healthy volunteers, showing a dose-dependent inhibition of collagen-induced platelet aggregation without affecting template bleeding times (70). A phase 2 randomized trial currently assessed the safety of glenzocimab infusion on the top of IV tPA in patients with AIS (ClinicalTrials.gov: NCT03803007).

Another strategy could be to administer a reversible antiplatelet drug with a short plasma half-life associated with early restoration of platelet function to prevent HT occurrence. This is now possible with cangrelor. This new platelet P2Y12 receptor inhibitor has a plasma half-life of about 5 min, and complete restoration of platelet aggregation is obtained approximately 1 h after the end of IV cangrelor infusion (71). A phase 3 randomized trial is currently assessing the benefit of cangrelor infusion in imaging-selected patients with AIS who were eligible for EVT (ClinicalTrials.gov: NCT04667078).

### vWF Multimers

The other strategy to enhance thrombolysis in AIS could be to target non-fibrin extracellular network components. Platelet cross-linking during thrombosis involves vWF multimers. Therefore, proteolysis of vWF multimers appears promising to disaggregate platelet-rich thrombi and restore vessel patency in AIS.

The first study from Denorme et al. (12) found that AIS thrombi contained about 20% of vWF. The authors suggested that targeting vWF with the specific vWF-cleaving metalloprotease ADAMTS13 could have a thrombolytic effect in an experimental thromboembolic model of AIS associated with reduced infarct volume (12).

More recently, a study assessed a potent thrombolytic effect of *N*-acetylcysteine (NAC, a clinically approved mucolytic drug). NAC has the ability to break up vWF multimers by reducing intrachain disulfide bonds in large polymeric proteins. This study found an increased recanalization rate after NAC vs. saline infusion, especially in the case of concomitant treatment with an αIIbβ3 inhibitor, which suggested a synergistic action between these two treatments (13). The NAC for thrombolysis in acute stroke (NAC-S) trial (ClinicalTrials.gov: NCT04920448) is an ongoing phase 2 randomized study that assesses the safety of NAC combined with IV tPA in an AIS setting.

### NETs Network

Three *ex vivo* studies found that recombinant DNAse 1 accelerated tPA-induced thrombolysis, whereas tPA alone was ineffective (7–9). These results indicate that co-administration of DNAse 1 and tPA could be of interest in the setting of AIS with large vessel occlusion. These results must be confirmed in future clinical studies. Recently, advances have been made regarding the role of DNase 1 as an adjuvant to tPA for thrombolysis of AIS thrombi. The ability of DNase 1 to improve tPA-mediated thrombolysis is linked to a potentiation of fibrinolysis, as indicated by increased release of plasmin-generated D-dimers in the presence of DNase 1 (44). As a result of these findings, a first clinical trial to assess the safety and efficacy of DNase 1 in patients with AIS who received IV tPA and are eligible for EVT (NETs-TARGET study, ClinicalTrials.gov: NCT04785066) will be launched in the coming months. Of note, the enzyme responsible for the NET formation, peptidylarginine deiminase type IV, was recently found released together with NETs into the extracellular milieu, thus leading to citrullination and inactivation of ADAMTS13 and impairing vWF multimer cleavage (72). This observation suggests that NETosis may result in increased resistance to not only tPA but also ADAMTS13. Therefore, peptidylarginine deiminase type IV inhibitors, several already developed (73), may be of clinical interest to improve the efficacy of thrombolytic cocktails combining tPA, ADAMTS13, and DNase 1.

## Conclusions and Perspectives

Future thrombolytic therapies for AIS will perhaps include the optimization of tPA therapy, but in our opinion, the most promising way will consist of targeting non-fibrin thrombus contents, especially their platelet-rich areas with aggregated platelets, vWF multimers, and NETs. This evidence supports a pharmacological cocktail for the future of AIS treatment that includes therapies targeting different contents of thrombi with synergistic actions. The development of such add-on therapies may represent unique opportunities to improve recanalization therapy and to reduce tPA doses and the associated risk of HT, which is responsible for increased mortality in patients with AIS who received tPA and in the end to individualize the acute recanalization treatment to the thrombus composition.

Whether thrombus composition can be deduced from circulating markers remains an open question. In fact, to date, if and how AIS thrombus composition relates to circulating levels of thromboinflammatory mediators (e.g., platelets, coagulation factors, neutrophils, NETs, vWF, and pro-inflammatory cytokines) has not been thoroughly investigated. Yet, in a prospective cohort study that included 131 consecutive EVT-treated patients with AIS, Prochazka et al. found a significant positive correlation between vWF plasma levels and thrombus vWF content (74). Moreover, there is accumulating evidence indicating that circulating markers of several thrombotic pathways are predictive of AIS outcomes in EVT-treated patients with AIS. Interestingly, these markers not only include vWF (74) but also other thrombus components, such as NETs, thus strengthening the notion that circulating and intra-thrombus markers may, at least in part, be interconnected. In particular, increased admission levels of circulating NETs markers (i.e., cell-free DNA and citrullinated histones) (75), increased vWF/ADAMTS13 ratio (74), or decreased plasma DNase (75) or ADAMTS13 activity (51, 76) have all been shown to be associated with unfavorable outcome in EVT-treated patients with AIS.

Although not the focus of the present review, it is worth mentioning that, in addition to acute recanalization therapies, insights from AIS thrombus composition analysis may also help to optimize secondary prevention strategies. In fact, results from several recent studies are converging to indicate that thrombus composition can provide information on stroke etiology, which is a major determinant of secondary prevention decisions. For example, we have shown recently on a limited patient with AIS cohort that intra-thrombus DNA content could help to reclassify ~50% of AIS patients with embolic stroke of undetermined source, into the cardioembolic subtype, with a specificity of 90% (77). Furthermore, considering the well-established pro-thrombotic role of NETs (43), the presence of extracellular DNA and NETs in AIS thrombi raises the question of whether DNase 1 should be considered in the future for improving secondary prevention therapies. Besides the therapeutic aspects, the fact that thrombus composition may, at least in part, reflect the etiology is also informative regarding the pathophysiology of AIS, as it suggests that some mechanisms (i.e., NETosis and immunothrombosis in general) may play a more prominent role in certain AIS subtypes.

## Author Contributions

J-PD, MM, and BH-T-N wrote the manuscript. All authors contributed to the article and approved the submitted version.

## Funding

This work was supported by INSERM, La Fondation pour la Recherche sur les AVC (grant # FR-AVC-003), La Fondation pour la Recherche Médicale (grant #DPC20171138959), La Fondation de l'Avenir (AP-RM-17-005), and by a public grant overseen by the French National Research Agency (ANR) as part of the Investments for the Future program (PIA) under grant agreement nos. ANR-18-RHUS-0001 (RHU Booster) and ANR-16-RHUS-0004 (RHU TRT_cSVD).

## Conflict of Interest

J-PD, MM, BH-T-N, MP, and LD are co-inventors of the following patent: Methods and pharmaceutical compositions for the treatment of acute ischemic stroke (PCT/EP2018/062588). The remaining authors declare that the research was conducted in the absence of any commercial or financial relationships that could be construed as a potential conflict of interest.

## Publisher's Note

All claims expressed in this article are solely those of the authors and do not necessarily represent those of their affiliated organizations, or those of the publisher, the editors and the reviewers. Any product that may be evaluated in this article, or claim that may be made by its manufacturer, is not guaranteed or endorsed by the publisher.
